# Distribution of Cytoskeletal Components in Endothelial Cells in the Guinea Pig Renal Artery

**DOI:** 10.1155/2012/439349

**Published:** 2012-03-05

**Authors:** Kazuo Katoh, Yasuko Noda

**Affiliations:** Department of Anatomy, Jichi Medical School, 3311-1 Yakushiji, Shimotsuke, Tochigi 329-0498, Japan

## Abstract

The cytoskeletal components of endothelial cells in the renal artery were examined by analysis of en face preparations under confocal laser scanning microscopy. Renal arterial endothelial cells were shown to be elongated along the direction of blood flow, while stress fibers ran perpendicular to the flow in the basal portion. Focal adhesions were observed along the stress fibers in dot-like configurations. On the other hand, stress fibers in the apical portion of cells ran along the direction of flow. The localizations of stress fibers and focal adhesions in endothelial cells in the renal artery differed from those of unperturbed aortic and venous endothelial cells. Tyrosine-phosphorylated proteins were mainly detected at the sites of cell-to-cell apposition, but not in focal adhesions. Pulsatile pressure and fluid shear stress applied over endothelial cells in the renal artery induce stress fiber organization and localization of focal adhesions. These observations suggest that the morphological alignment of endothelial cells along the direction of blood flow and the organization of cytoskeletal components are independently regulated.

## 1. Introduction

The cytoskeletal components of endothelial cells play important roles in maintaining the fundamental structure of the thin inner layer of the blood vessels called the endothelium. Endothelial cells form a single cell layer on the surfaces of blood vessels and are therefore constantly subjected to both fluid shear stress and periodic strain caused by blood pressure induced by the pulsatile flow. Endothelial cells have been shown to possess many stress fibers both *in vitro *and *in situ*, and these cells are known to be capable of responding to the level of fluid shear stress by changing their shape [1, 2], distribution of cytoskeletal components [3–5], expression of signal transduction-associated proteins [6, 7], and expression of various genes [8, 9]. Endothelial cells in culture are known to respond to cyclic stretching [10] and hyperosmotic shock [7]. On exposure to uniaxial cyclic stretching, endothelial cells become aligned perpendicular to the axis of stretching [10, 11]. Cyclic stretching applied to cells in culture is a model of pulsatile stretching induced by blood pressure *in vivo,* and the effects differ from those of fluid shear stress generated by the blood flow. Cyclic stretching is experienced by both the apical and basal portions of the cell. Hemodynamic shear stress caused by blood flow occurs in combination with cyclic stretching caused by the pressure of pulsatile flow generated by blood pressure.

Previous *in vivo *experiments indicated that stress fibers in endothelial cells respond to fluid shear stress and show increases in both number and thickness in a manner related to the magnitude of shear stress (for review, see Katoh et al., 2008) [12]. Previously, we reported increases in number and thickness of stress fibers and focal adhesions in both the apical and basal portions of endothelial cells in an artificial coarctation zone in the abdominal aorta where fluid shear stress is significantly high in comparison to endothelial cells subjected to averaged shear stress [6]. The plaque-like vinculin-containing spots detected at the ends of stress fibers were enlarged in the coarctation area, especially in the apical portions of the cells [6]. In addition, stress fibers and their sites of association with the plasma membrane are closely attached to both the apical and basal portions of endothelial cells, and we suggested that they may play key roles in force transfer by fluid stress [13, 14]. Our findings also suggested that even in the case of endothelial cells* in situ*, the apical plaques (i.e., stress fiber-plasma membrane attachment sites with accumulation of focal adhesion-associated proteins) and their associated stress fibers are candidates for sensing and/or transferring mechanical signals of fluid shear stress applied to the laminar surface of endothelial cells [6, 14]. Apical plaques are enlarged in the apical portion of endothelial cells in the coarctation zone reflecting the response of the apical plaque and its associated stress fibers to mechanical stimuli generated by blood flow [6]. Such responses increase according to the magnitude of applied shear stress, in agreement with the observations in traditional *in vitro *cell culture systems [2, 15–18].

Stress fibers are major higher-order structural components of the cytoskeleton in nonmuscle cells, which are composed of actomyosin filaments and show contractility both *in vitro *[19] and *in situ* [20]. We reported previously that stress fibers could be isolated from fibroblasts without loss of morphological or functional characteristics and that they represent a major part of the contractile apparatus within the cell [19]. The principal role of stress fibers is related to their contractility within the cell. We also reported that the stress fibers are located not only in the basal portion of the cell, but also in the apical portion in both cultured fibroblasts [13] and in guinea pig aortic endothelial cells [14], and we called these apically located stress fibers “apical stress fibers.” Some apical stress fibers not only connect to the apical plaques but also make direct connections with focal adhesions in the basal portion of endothelial cells, and the apical stress fibers have the ability to transfer mechanical forces from the apical to the basal portion of the cell. Apical stress fibers in endothelial cells* in situ* are directly subjected to fluid shear stress, and the mechanical stimuli generated by this fluid shear stress are applied directly to the apical stress fibers.

Blood vessels in the living animal are subjected to pulsatile stretches generated by the heart *via *the circulatory system. These pulsatile stretches seem to induce changes in endothelial cell shape and the formation of cytoskeletal components. Previous *in vitro *experiments showed that cyclic stretching applied to cells in culture causes the cells to become oriented perpendicular to the direction of stretching [10, 21, 22] consistent with *in vivo *results [23]. On the other hand, in cells exposed to unidirectional tension, the stress fibers become organized along the axis of tension [24].

*In situ *experiments indicated that guinea pig venous endothelial cells were elongated in the direction of blood flow to a greater extent than unperturbed aortic endothelial cells [25]. Moreover, thick stress fibers located at the basal side of venous endothelial cells were fewer in number than in aortic endothelial cells. The morphological differences between venous and aortic endothelial cells seem to be due to the sustained exposure of the former cell type to significantly lower levels of fluid shear stress than the latter. However, cell culture conditions preclude accurate observations because the cells have been artificially removed from the living animal. Therefore, analyses of the fundamental mechanisms involved in the responses to mechanical stimuli, such as fluid shear stress, pulsatile enlargement of blood vessel diameter, and/or stretching, should be performed in living intact blood vessels.

In the basal portion of endothelial cells, stress fibers generally run along the axis of blood flow in typical aortic and venous endothelial cells. However, we reported previously that stress fibers in the apical portion of venous endothelial cells run perpendicular to the direction of blood flow [25]. These observations raised questions regarding whether the mechanism by which stress fibers run is independent of the direction of blood flow. Both the right and left renal arteries branch off from the abdominal aorta at an angle of 90° and carry blood to the kidneys. Approximately 1/3 of the blood from the heart is directed into the kidneys. Blood in the renal artery is filtered by the kidneys, and so the resistance to blood flow applied to the surface of the renal artery should be higher than that in most other arteries. Mechanical stress applied to the endothelial cells in the renal artery should be different from the straight portion of the abdominal aorta *in situ*, and therefore the distribution of cytoskeletal components, such as stress fibers and focal adhesions, should differ considerably between renal artery endothelial cells and endothelial cells experiencing unidirectional flow *in situ*. The observations outlined above prompted us to examine the detailed distributions of cytoskeletal components and associated proteins. Here, we carefully compared the cytoskeletal components of endothelial cells in the renal artery with those of unperturbed arterial and venous endothelial cells. The results indicated that the cytoskeletal components of endothelial cells in the renal artery showed quiet different distribution patterns from the stress fibers in unperturbed aortic and venous endothelial cells.

## 2. Materials and Methods

### 2.1. Preparation of Blood Vessels

Aortae were obtained from adult guinea pigs weighing 250–400 g. Some animals were treated as described below. All animals were anesthetized with an overdose of sodium pentobarbital administered by intraperitoneal injection. After perfusion with 0.85% NaCl containing 200 units of heparin sodium *via *the left ventricle, the abdominal aortae were excised and fixed with 1% or 2% paraformaldehyde using the microwave irradiation method [26].

### 2.2. Antibodies and Fluorescent Reagents

Monoclonal antivinculin (Sigma, St. Louis, MO), antipaxillin (BD Transduction Laboratories, San Jose, CA), anti-alpha-actinin (Sigma), and antiphosphorylated tyrosine-containing protein antibody (clone PY-20; ICN Pharmaceuticals, Aurora, OH) were purchased from the sources indicated. FITC-labeled secondary antibody against rabbit IgG was purchased from Zymed (San Francisco, CA). Rhodamine-labeled phalloidin was purchased from Molecular Probes (Eugene, OR).

### 2.3. Immunofluorescence Procedures and Observation by Confocal Laser Scanning Microscopy

Fixed aortae were rinsed several times with phosphate-buffered saline (PBS) and cut into small pieces. After permeabilization with 0.5% Triton X-100 in PBS for 5 min, specimens were incubated with antibodies; some specimens were double-stained with rhodamine-labeled phalloidin. Some specimens were stained using microwave irradiation method [26, 27]. Specimens were mounted in 90% glycerol in PBS containing 2.5% 1,4-diazabicyclo[2.2.2]octane (DABCO; Sigma) and examined by confocal laser scanning microscopy (CLSM) (FV-1000; Olympus, Tokyo, Japan) with a Plan Apochromat ×60 (N.A. 1.4, oil) objective lens (Olympus). Stereo pair images were reconstructed from 20 to 30 serial optical sections obtained by CLSM using IMAGE J software (NIH, Bethesda, ML).

### 2.4. Measurement of Renal Artery Blood Flow

For ultrasound microimaging, renal arteries were obtained from adult guinea pigs weighing approximately 380–420 g. Ultrasound images of the renal artery were obtained using a high-frequency ultrasound imaging system (Vevo 770; VisualSonics, Toronto, ON, Canada). Animals were anesthetized by intraperitoneal injection of pentobarbital sodium at a dose of 10 mg/400 g body weight (Abbott Laboratories, Abbott Park, IL). After perfusion, the abdomen was surgically opened, and living ultrasound images of the renal artery and abdominal aorta were obtained, and blood flow speed was measured using an ultrasound imaging system.

## 3. Results

### 3.1. Distribution of Stress Fibers and Focal Adhesions in Endothelial Cells in the Renal Artery

**Figure 1:**
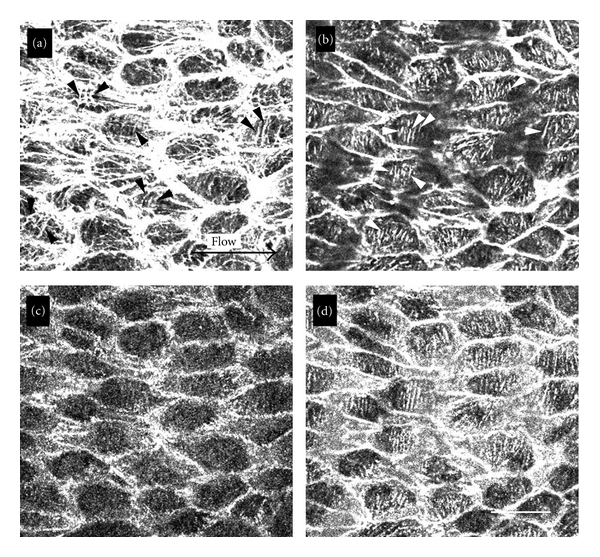
En face preparations of the renal artery were double-stained with rhodamine-labeled phalloidin (a and b) and antivinculin antibody (c and d). Focus was adjusted to apical (a and c) and basal (b and d) portions of the endothelial cells. In the apical portion of the cells, several stress fibers running perpendicular to the direction of flow were observed (a: arrowheads) among the stress fibers running parallel to the direction of flow (a: arrow). Only stress fibers running perpendicular to the direction of blood flow were seen in the basal portion of the cells (b: arrowheads). All spots showing positive labeling with antivinculin antibody were colocalized with stress fibers (d). Bar: 100 *μ*m.

**Figure 2:**
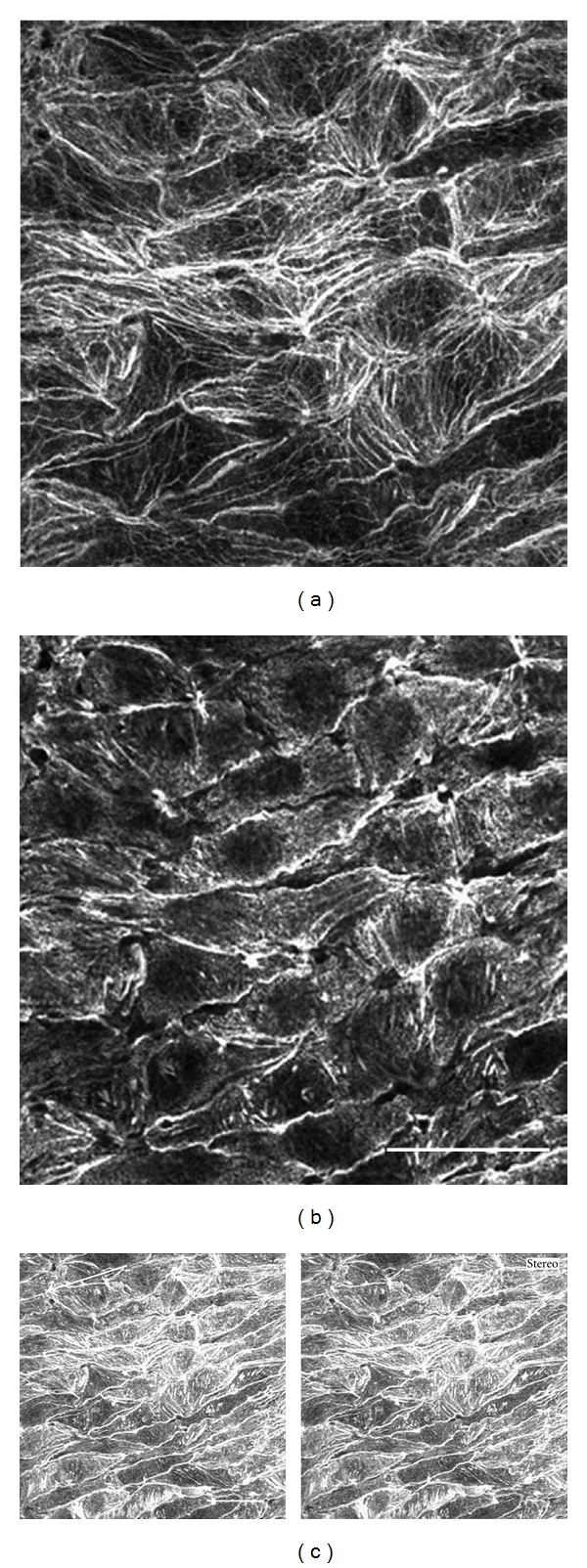
Endothelial cells in the renal artery stained with rhodamine-labeled phalloidin. The focal plane was adjusted to the surface (a) or basal (b) portion of the endothelial cells. The three-dimensional distribution of actin filaments reconstructed using 20 serial confocal optical sections from (a) to (b) is shown in (c) (stereo pair image). Thick stress fibers were observed in the basal portion of the cells oriented perpendicular to the direction of blood flow (b: see also stereo pair image). In contrast, both parallel and perpendicular stress fibers oriented along the direction of flow were seen in the apical portion of the cells (a: see also stereo pair image). To see stereo image (c), use special glasses or a light source split into the viewer's eyes. The focal plane closer to the viewer in stereo image (c) is the apical surface of the renal endothelial cells. The arrow indicates the direction of blood flow. Bars in (b) and (c): 100 *μ*m.

The distributions of stress fibers and focal adhesions were determined by confocal laser scanning microscopy in whole-mount preparations of the guinea pig renal artery. Specimens were double stained with rhodamine-labeled phalloidin for visualization of stress fibers and with antivinculin antibody for visualization of focal adhesions (Figure 1). Phalloidin-positive actin-containing filaments in the renal artery also showed a punctate linear staining pattern with anti-alpha-actinin antibody, so they were classified as well-developed stress fibers (data not shown). When the focal plane was adjusted at the basal portion of the cell, stress fibers were shown to run perpendicular to the direction of blood flow (Figure 1(b); arrowheads). On the other hand, when the focal plane was adjusted to the apical portion of the endothelial cells, many stress fibers were observed running along the direction of blood flow (Figure 1(a); flow). Some stress fibers were also shown to run perpendicular to the direction of flow (Figure 1(a); arrowheads). Both ends of the stress fibers were associated with vinculin (Figure 1(d)) or paxillin (data not shown), confirming the identity of vinculin-positive spots as focal adhesions. Focal adhesions revealed by staining with antivinculin antibody were closely associated with the stress fibers in the basal portion of the cell in a dot-like configuration (Figure 1(d)). Significant immunofluorescent staining with antivinculin antibody was not detected at the apical surface of the cells. However, intense staining was also detected at the sites of cell-to-cell association, presumably adherens junctions of the endothelial cells (Figure 1(c)). Stress fibers in the unperturbed aortic endothelial cells ran along the direction of blood flow in the basal portion of the cell [14, 28]. In the basal portion of the cell, the stress fibers in the renal artery were shown to run perpendicular to the direction of blood flow. In the apical portion of unperturbed endothelial cells located in the abdominal aorta, stress fibers were only observed along the direction of blood flow in both the apical and basal portions [14]. To determine the distribution of stress fibers in renal arterial endothelial cells in greater detail, three-dimensional stereo images stained with rhodamine-labeled phalloidin were reconstructed from confocal serial optical sections (Figure 2(c) for stereo image). Many stress fibers running both parallel and perpendicular to the direction of flow were observed in the apical portion of the cell (Figure 2(a) for a single confocal image). Only thick stress fibers running perpendicular to the direction of blood flow were observed in the basal portion of the cell (Figure 2(b) for a single confocal image; Figure 2(c) for stereo image).

### 3.2. Distribution of Tyrosine-Phosphorylated Proteins in Endothelial Cells in the Renal Artery

**Figure 3:**
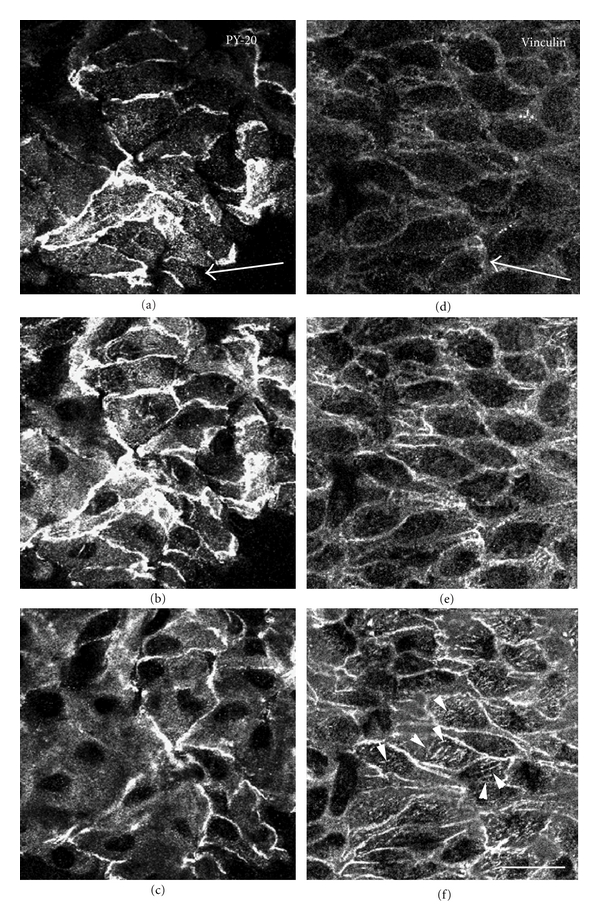
Localization of tyrosine-phosphorylated proteins and vinculin in the renal arterial endothelial cells. En face preparations were stained with antiphosphotyrosine antibody (PY-20) (a–c) or antivinculin antibody (d–f) and observed by confocal laser scanning microscopy. The focal plane was adjusted to the surface (a and d), middle (b and e), or basal (c and f) portion of the endothelial cells. Tyrosine-phosphorylated proteins were observed along the apical plasma membrane in uniform staining (a). Strong staining for tyrosine-phosphorylated proteins was detected at sites of cell-to-cell apposition (b), with faint staining in the cytoplasm (c). Antivinculin staining was detected at sites of cell-to-cell apposition (e) and focal adhesion in the basal portion of the cells (f: arrowheads). No staining for vinculin was detected at the apical surface of the cells (d). The arrow indicates the direction of blood flow. Bar: 50 *μ*m.

**Figure 4:**
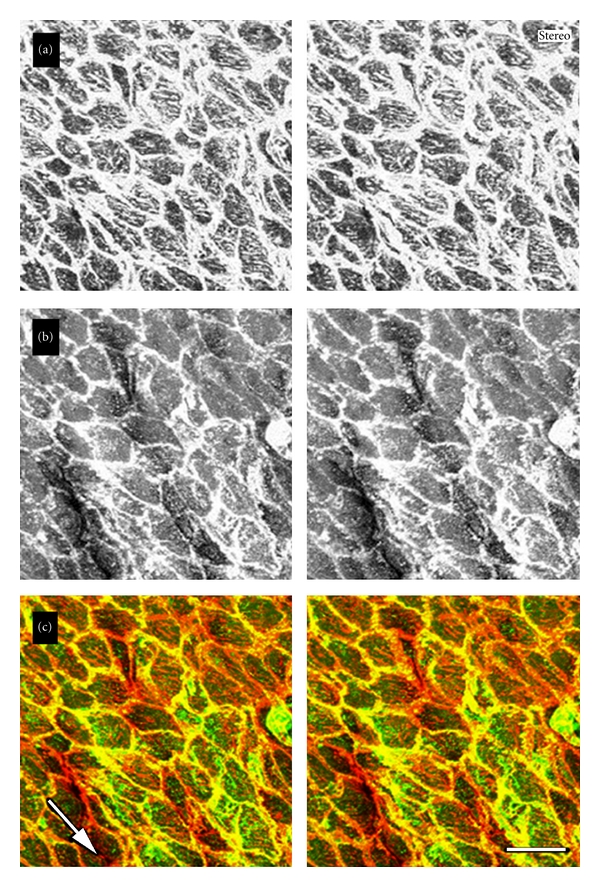
Stereo pair of endothelial cells in the renal artery double-stained with rhodamine-labeled phalloidin (a) and antiphosphotyrosine antibody (PY-20) (b). A merged image (a and b) is shown in (c). The three-dimensional distributions of actin filaments (c: red) and tyrosine-phosphorylated proteins (c: green) are shown. The region of overlap between actin filaments and tyrosine-phosphorylated protein is shown in yellow (c). Tyrosine-phosphorylated proteins were observed below the plasma membrane, mainly at sites of cell-to-cell apposition and on the apical surface of the endothelial cells. To see the stereo image, use special glasses or a light source split into the viewer's eyes. The focal plane closer to the viewer is the apical surface of the renal endothelial cells. The arrow indicates the direction of blood flow. Bar: 50 *μ*m.

As certain proteins undergo tyrosine phosphorylation during intracellular signaling events, we next examined the tyrosine phosphorylation levels of endothelial cells in the renal artery (Figure 3). En face preparations stained with an antibody against phosphotyrosine-containing proteins or an antibody against vinculin were examined by confocal laser scanning microscopy. Positive staining with antiphosphotyrosine antibody was observed in a uniform pattern at the apical surface of the endothelial cells (Figure 3(a)). When the focal plane was adjusted to the middle level of the cells, staining was detected at sites of cell-to-cell apposition (Figure 3(b)). The focal plane was adjusted to the basal portion of the cell, and staining with antiphosphotyrosine antibody was mainly detected at cell-to-cell adhesion sites, but not along stress fibers or focal adhesions (Figure 3(c)). On the other hand, staining with antivinculin was detected at both sites of cell-to-cell apposition (Figure 3(e)) and focal adhesions at the basal side of the cell (Figure 3(f)). No significant staining was detected at the apical surface of the cell (Figure 3(d)). Staining with antivinculin and antiphosphotyrosine antibodies did not show complete colocalization at the basal portion of cells (Figures 3(c) and 3(f)). The renal artery was also double-stained for F-actin with rhodamine-labeled phalloidin and with antiphosphotyrosine antibody.  Figure 4 shows a stereo view of a cell double-stained with rhodamine-labeled phalloidin to visualize actin filaments and with antiphosphotyrosine antibody, and the results indicated no colocalization of tyrosine-phosphorylated proteins and stress fibers. Positive staining for phosphotyrosine was detected on the plasma membrane over endothelial cells, especially along the cell-to-cell adhesion sites. However, the specific distribution of staining for phosphotyrosine was not detected along the stress fibers in the basal or apical portions of the cell.

### 3.3. Imaging and Measurement of Blood Flow in the Renal Artery with an Ultrasound Microimaging System

**Figure 5:**
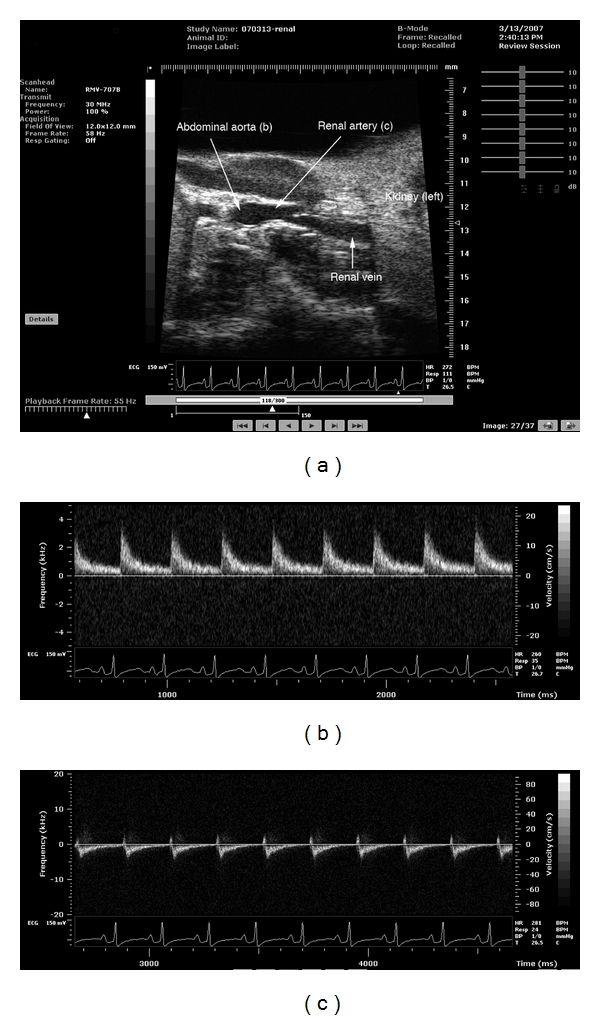
Ultrasound microimaging around the left kidney (a) and Doppler flow measurement at the abdominal aorta (b) and renal artery (c). Appropriate positions of the abdominal aorta, renal artery, and renal vein observed with the ultrasound imaging device are indicated in (a). The blood flow of the abdominal aorta indicated by the arrow in (a) is shown in (b). The blood flow of the renal artery indicated by the arrow in (a) is shown in (c). Doppler flow data show unidirectional (b) and oscillating (c) flow patterns in the abdominal aorta and renal artery, respectively. Note that the waveform of the renal artery indicated in (c) is the opposite of that for the abdominal aorta in (b) because of the position of the ultrasound probe.

To visualize and measure blood flow in the renal artery, we measured biological parameters using an ultrasound microimaging system. Living images of the abdominal aorta and the renal artery are shown in Figure 5(a). Doppler blood flow recordings were also made for the abdominal aorta (Figure 5(b)) and renal artery (Figure 5(c)). Retrograde blood flow was detected in the renal artery in cyclic diastolic phases (Figure 5(c)), indicating that the renal arterial endothelial cells are subjected to forward and backward force caused by the circulatory pulse. It is difficult to calculate the precise shear stress applied to endothelial cells in the renal artery because of the complicated forward and backward flow, pulsatile pressure, and resistance pressure from the kidney. However, pulsatile flow in the renal artery results in significant fluctuations in the magnitude of shear stress and/or pressure, which are not predicted to occur in unperturbed aortic endothelial cells. Further studies are required to precisely determine the fluid shear stress and pressure applied to the endothelial cells in the renal artery. Fluctuating and retrograde flow in the renal artery seem to have an influence on stress fiber localization, as stress fibers run perpendicular to the direction of blood flow in the basal portion of the cells (see Figures 1 and 2).

## 4. Discussion

Endothelial cells respond to fluid shear stress and show changes in morphology and organization of cytoskeletal components. Unperturbed aortic endothelial cells, such as those in the straight portion of the abdominal aorta, show elongation along the direction of blood flow. In this region, cytoskeletal components, such as stress fibers and focal adhesions, are also enhanced in a manner corresponding to the magnitude of blood flow [6, 14]. We reported previously that cytoskeletal components, such as stress fibers and focal adhesions, are significantly increased in both number and size in the surgical coarctation zone in the straight portion of the abdominal aorta where fluid shear stress is expected to be high [6]. Confocal laser scanning microscopy indicated the presence of thick stress fibers in the basal portion of the cells in the artificial coarctation zone [6]. Stress fibers were also observed in the apical portion along the direction of blood flow, where they are continually exposed to mechanical stimulation generated by the blood flow. On the other hand, venous endothelial cells had fewer thick basal stress fibers than unperturbed aortic endothelial cells [25]. Moreover, many apical stress fibers running perpendicular to the direction of blood flow were seen in the apical region of venous epithelial cells [25]. The results of the present study indicated that stress fibers run perpendicular to the direction of blood flow in both the apical and basal regions of renal endothelial cells. Focal adhesions were also colocalized with stress fibers in these cells. Although the renal arterial endothelial cells were elongated along the direction of flow, stress fibers were localized perpendicular to the axis of elongation in the basal portion of these cells. This was different from the distribution of stress fibers in unperturbed endothelial cells. The complex nature of the different environmental conditions seemed to induce alterations in the cytoskeletal components and associated morphological changes in these cells, compared to unperturbed aortic and venous endothelial cells. High fluid shear stress is generally thought to result in increases in both the number and thickness of stress and focal adhesions. However, the results of the present study indicated that the arrangement of stress fibers and cell shape along the direction of blood flow were independently regulated by mechanical stimuli. Fluid shear stress induced orientation of the stress fibers and associated focal adhesions along the direction of blood flow, while oscillatory flow caused the stress fibers and focal adhesions to be oriented perpendicular to the direction of blood flow.  Table 1 summarizes the directions of stress fibers in aortic, venous, and renal arterial endothelial cells.

Certain proteins undergo tyrosine phosphorylation during intracellular signaling events. Here, we showed that tyrosine-phosphorylated proteins are localized at the endothelial cell surface and sites of cell-to-cell apposition in the renal artery. These observations were consistent with those in unperturbed aortic endothelial cells [14], so that signaling by mechanical stimuli may occur in the same position in renal arterial and other arterial endothelial cells *in situ*. We and other groups have previously identified several proteins that undergo tyrosine phosphorylation induced by fluid shear stress. PECAM-1, which is mainly localized at the sites of endothelial cell-to-cell apposition, is tyrosine phosphorylated when endothelial cells are subjected to high levels of shear stress [7]. Src family proteins are also tyrosine phosphorylated when cells are subjected to high shear stress [29]. Moreover, expression of c-Src is increased in the artificial coarctation zone in the abdominal aorta [6]. These observations suggested that PECAM-1 and c-Src are primary candidates for signal transduction of mechanical stimuli in endothelial cells in the renal artery. The bidirectionality of the oscillating blood flow itself may be the key to the perpendicular orientation, or some unique protein(s) made only by renal endothelial cells may interact to perpendicularly reorient the basal stress fibers. We are currently engaged in studies of the unique molecules expressed in renal arterial endothelial cells.

Stress fibers running perpendicular to the direction of blood flow were shown to be present at (1) the apical surface of venous endothelial cells [25], (2) the apical surface of aortic endothelial cells in the artificial coarctation zone [6], (3) the apical side of renal arterial endothelial cells (as demonstrated in the present study), and (4) the basal side of renal arterial endothelial cells (as demonstrated in the present study). The directions of stress fibers in endothelial cells in aortic, venous, and renal arteries are summarized in Table 1. Venous endothelial cells also possess apical stress fibers that run perpendicular to the direction of blood flow. However, in the basal portion of venous endothelial cells, stress fibers were found to run along the direction of flow. Although the reasons for the above discrepancy related to blood flow are still unclear, it is likely that the stress fiber direction is determined not only by the direction of blood flow but also by other mechanical forces, such as pressure and/or stretching force to which the endothelial cells are exposed.

It is of Interest that endothelial cells in the renal artery were aligned along the direction of blood flow. In the basal portions of the cell, all of the stress fibers ran perpendicular to the direction of blood flow. Moreover, focal adhesions in this region were also aligned along the perpendicular stress fibers. The alignments of stress fibers and focal adhesions in this region were completely independent of the direction of blood flow. On the other hand, in the apical portion of the renal arterial endothelial cells, stress fibers were aligned along the direction of flow. The mechanism underlying the organization of stress fibers that run perpendicular to the direction of blood flow is unclear, but it is likely that these “perpendicular” stress fibers respond to stretching force generated by blood pressure applied to the endothelial cells. In mesothelial cells subjected to single-direction stretching, stress fibers were aligned in the direction of stretch [24]. On the other hand, cells subjected to cyclic stretching became aligned perpendicular to the direction of stretching force [23]. Stretch-activated (SA) ion channels play a role in reorientation of endothelial cells that is not dependent on fluid shear stress force. An inhibitor of SA channels was shown to block SA-induced reorientation of endothelial cells, but no changes were observed in stress fiber organization by the inhibitor [30]. These observations suggest that the stress fibers running perpendicular to the direction of flow may be organized by the stretching force generated by blood flow.

In the apical portion of the cell, stress fibers were found to run along the direction of blood flow as observed in unperturbed aortic endothelial cells. These observations suggested that the orientation of apical stress fibers was primarily induced by the fluid shear stress, because the endothelial cell surface is directly exposed to fluid shear stress together with pulsatile pressure. The organization of cytoskeletal components in endothelial cells on the inner surface of blood vessels in the renal artery is controlled by local mechanical stimuli, consisting of a complex combination of bidirectional fluid shear stress, and oscillatory longitudinal and/or circumferential pressure. In this study, we found a difference between the directionality of stress fibers in renal endothelial cells compared to the unperturbed aortic endothelial cells. This difference may be due to the different environmental conditions. The renal artery branches off from the abdominal aorta at an angle of almost 90° and is inserted directly into the kidney. This geometry may result in special physiological blood flow conditions, such as stretching force over the endothelial cells. The morphological alignment of endothelial cells along the direction of blood flow and the organization of cytoskeletal components were shown to be independently regulated. Further studies are required to gain insight into the organization of cytoskeletal components and associated changes in cell shape in response to mechanical stimuli applied to endothelial cells in both* in situ *and* in vitro*.

## Figures and Tables

**Table 1:** Orientations of stress fibers in aortic, venous, and renal arterial endothelial cells.

	Apical		Basal	
	Parallel	Perpendicular	Parallel	Perpendicular
Aorta	+	−	++	−
Venous	−	+	+	−
Renal artery	+	+	−	++

Parallel: stress fibers oriented parallel to the direction of blood flow. Apical: stress fibers localized to the apical portion of the endothelial cells. Basal: stress fibers localized to the basal portion of the endothelial cells. Perpendicular: stress fibers oriented perpendicular to the direction of blood flow.

–: no stress fibers were detected; +: thin stress fibers were detected; ++: thick stress fibers were detected.

Aorta: endothelial cells in the straight portion of the abdominal aorta. Venous: endothelial cells in the straight portion of the inferior vena cava.

*See Katoh et al., 2007 [25].

## References

[1] Flaherty JT, Pierce JE, Ferrans VJ, Patel DJ, Tucker WK, Fry DL (1972). Endothelial nuclear patterns in the canine arterial tree with particular reference to hemodynamic events. *Circulation Research*.

[2] Dewey CF, Bussolari SR, Gimbrone MA, Davies PF (1981). The dynamic response of vascular endothelial cells to fluid shear stress. *Journal of Biomechanical Engineering*.

[3] White GE, Gimbrone MA, Fujiwara K (1983). Factors influencing the expression of stress fibers in vascular endothelial cells in situ. *Journal of Cell Biology*.

[4] Franke RP, Grafe M, Schnittler H (1984). Induction of human vascular endothelial stress fibres by fluid shear stress. *Nature*.

[5] Wong AJ, Pollard TD, Herman M (1983). Actin filament stress fibers in vascular endothelial cells in vivo. *Science*.

[6] Kano Y, Katoh K, Fujiwara K (2000). Lateral zone of cell-cell adhesion as the major fluid shear stress- related signal transduction site. *Circulation Research*.

[7] Osawa M, Masuda M, Kusano KI, Fujiwara K (2002). Evidence for a role of platelet endothelial cell adhesion molecule-1 in endothelial cell mechanosignal transduction: is it a mechanoresponsive molecule?. *Journal of Cell Biology*.

[8] Davies PF (1995). Flow-mediated endothelial mechanotransduction. *Physiological Reviews*.

[9] Obi S, Yamamoto K, Shimizu N (2009). Fluid shear stress induces arterial differentiation of endothelial progenitor cells. *Journal of Applied Physiology*.

[10] Sai X, Naruse K, Sokabe M (1999). Activation of pp60(src) is critical for stretch-induced orienting response in fibroblasts. *Journal of Cell Science*.

[11] Kanda K, Matsuda T (1993). Behavior of arterial wall cells cultured on periodically stretched substrates. *Cell Transplantation*.

[12] Katoh K, Kano Y, Ookawara S (2008). Role of stress fibers and focal adhesions as a mediator for mechano-signal transduction in endothelial cells in situ. *Vascular Health and Risk Management*.

[13] Katoh K, Masuda M, Kano Y, Jinguji Y, Fujiwara K (1995). Focal adhesion proteins associated with apical stress fibers of human fibroblasts. *Cell Motility and the Cytoskeleton*.

[14] Kano Y, Katoh K, Masuda M, Fujiwara K (1996). Macromolecular composition of stress fiber-plasma membrane attachment sites in endothelial cells in situ. *Circulation Research*.

[15] Eskin SG, Ives CL, McIntire LV, Navarro LT (1984). Response of cultured endothelial cells to steady flow. *Microvascular Research*.

[16] Reidy MA, Langille BL (1980). The effect of local blood flow patterns on endothelial cell morphology. *Experimental and Molecular Pathology*.

[17] Nerem RM, Levesque MJ, Cornhill JF (1981). Vascular endothelial morphology as an indicator of the pattern of blood flow. *Journal of Biomechanical Engineering*.

[18] Yoshida Y, Okano M, Wang S (1995). Hemodynamic-force-induced difference of interendothelial junctional complexes. *Annals of the New York Academy of Sciences*.

[19] Katoh K, Kano Y, Masuda M, Onishi H, Fujiwara K (1998). Isolation and contraction of the stress fiber. *Molecular Biology of the Cell*.

[20] Murakami T, Ishikawa H (1991). Stress fibers in situ in proximal tubules of the rat kidney. *Cell Structure and Function*.

[21] Shirinsky VP, Antonov AS, Birukov KG (1989). Mechano-chemical control of human endothelium orientation and size. *Journal of Cell Biology*.

[22] Takemasa T, Sugimoto K, Yamashita K (1997). Amplitude-dependent stress fiber reorientation in early response to cyclic strain. *Experimental Cell Research*.

[23] Sipkema P, Van Der Linden PJW, Westerhof N, Yin FCP (2003). Effect of cyclic axial stretch of rat arteries on endothelial cytoskeletal morphology and vascular reactivity. *Journal of Biomechanics*.

[24] Sugimoto K, Fujii S, Yamashita K (1991). Expression of stress fibers in bullfrog mesothelial cells in response to tension. *Experimental Cell Research*.

[25] Katoh K, Kano Y, Ookawara S (2007). Morphological differences between guinea pig aortic and venous endothelial cells in situ. *Cell Biology International*.

[26] Katoh K, Kano Y, Ookawara S (2009). Microwave irradiation for fixation and immunostaining of endothelial cells in situ. *Biotechnic and Histochemistry*.

[27] Katoh K (2011). Rapid fixation and immunofluorescent staining of cultured cells using microwave irradiation. *Journal of Histotechnology*.

[28] Kim DW, Langille BL, Wong MKK, Gotlieb AI (1989). Patterns of endothelial microfilament distribution in the rabbit aorta in situ. *Circulation Research*.

[29] Jalali S, Li YS, Sotoudeh M (1998). Shear stress activates p60src-Ras-MAPK signaling pathways in vascular endothelial cells. *Arteriosclerosis, Thrombosis, and Vascular Biology*.

[30] Hayakawa K, Sato N, Obinata T (2001). Dynamic reorientation of cultured cells and stress fibers under mechanical stress from periodic stretching. *Experimental Cell Research*.

